# The impact of platelets on pulmonary microcirculation throughout COVID-19 and its persistent activating factors

**DOI:** 10.3389/fimmu.2022.955654

**Published:** 2022-09-28

**Authors:** Mengqi Xiang, Xiaoming Wu, Haijiao Jing, Langjiao Liu, Chunxu Wang, Yufeng Wang, Valerie A. Novakovic, Jialan Shi

**Affiliations:** ^1^ Department of Hematology, the First Hospital, Harbin Medical University, Harbin, China; ^2^ Department of Research, Veterans Affairs (VA) Boston Healthcare System, Harvard Medical School, Boston, MA, United States; ^3^ Department of Medical Oncology, Dana-Farber Cancer Institute, Harvard Medical School, Boston, MA, United States

**Keywords:** COVID-19, platelet, microvesicles, phosphatidylserine, antiplatelet, thrombosis

## Abstract

Patients with COVID-19 often have hypoxemia, impaired lung function, and abnormal imaging manifestations in acute and convalescent stages. Alveolar inflammation, pulmonary vasculitis, and thromboembolism synergistically damage the blood-air barrier, resulting in increased pulmonary permeability and gas exchange disorders. The incidence of low platelet counts correlates with disease severity. Platelets are also involved in the impairment of pulmonary microcirculation leading to abnormal lung function at different phases of COVID-19. Activated platelets lose the ability to protect the integrity of blood vessel walls, increasing the permeability of pulmonary microvasculature. High levels of platelet activation markers are observed in both mild and severe cases, short and long term. Therefore, the risk of thrombotic events may always be present. Vascular endothelial injury, immune cells, inflammatory mediators, and hypoxia participate in the high reactivity and aggregation of platelets in various ways. Microvesicles, phosphatidylserine (PS), platelets, and coagulation factors are closely related. The release of various cell-derived microvesicles can be detected in COVID-19 patients. In addition to providing a phospholipid surface for the synthesis of intrinsic factor Xase complex and prothrombinase complex, exposed PS also promotes the decryption of tissue factor (TF) which then promotes coagulant activity by complexing with factor VIIa to activate factor X. The treatment of COVID-19 hypercoagulability and thrombosis still focuses on early intervention. Antiplatelet therapy plays a role in relieving the disease, inhibiting the formation of the hypercoagulable state, reducing thrombotic events and mortality, and improving sequelae. PS can be another potential target for the inhibition of hypercoagulable states.

## Introduction

Many COVID-19 patients, in both acute and convalescent stages, have symptoms of shortness of breath and dyspnea, accompanied by hypoxemia, impaired lung function (the diffusing capacity of the lung for carbon monoxide (DLCO) <80%), and abnormal imaging manifestations (interstitial infiltration, ground-glass opacity (GGO) and fibrosis) (1–5). Pulmonary diffusing dysfunction is ultimately attributed to damage to the blood-air barrier, including abnormalities in alveolar ventilation and pulmonary blood perfusion. Alveolar inflammation, pulmonary vasculitis, and thrombosis/embolism all have detrimental effects on pulmonary alveoli and microcirculation. Under the migration of macrophages that exist in the alveolar interstitium, pulmonary inflammation first appears on the alveolar side of the blood-air barrier. Further infection of type II alveolar epithelial cells by a large number of viruses leads to various immune cells, inflammatory mediators, and cytokines interacting to initiate inflammatory cascades, forming a cytokine storm and allowing more extensive infiltration of the alveoli (6, 7). In severe cases, the immune system is compromised, and the population of lymphocytes decreases or even collapses. The immune response can spread to vascular endothelial cells, allowing SARS-CoV-2 to directly invade angiotensin-converting enzyme 2 (ACE2) receptor-expressing vascular endothelial cells. Local vasoconstriction occurs after pulmonary vasculitis. The exposed collagen at the injury site promotes platelet activation, initiates the coagulation cascade, and eventually forms microthrombi or large thrombi in situ, resulting in increased intravascular pressure (8, 9). The mechanism by which platelets lead to impaired lung function has not been elucidated in detail. The function of platelets is complex. Platelet count and activity may be abnormal during both the acute and convalescent phases of COVID-19. The influence of virus-induced microenvironmental changes which induce platelets into a procoagulant phenotype appear to exist during many stages of COVID-19. COVID-19 patients have been shown to exhibit high levels of platelet-derived microvesicles (pEVs), similar to patients with acute respiratory distress syndrome (ARDS) and H1N1. Therefore, this review focuses on the effect of platelets on pulmonary microcirculation (including vascular endothelial injury and thrombosis) and explores the capability of endothelial injury, inflammation, and hypoxia to promote the formation of procoagulant platelets throughout COVID-19 disease progression. Additionally, we emphasize the role of phosphatidylserine (PS)-positive microvesicles (MVs) and discuss the treatment for COVID-19 hypercoagulability and thrombosis.

## Low platelet count: Weak endothelial protection

In SARS-CoV-2 infection, platelet consumption induced by pulmonary or extrapulmonary thrombosis/embolism and platelet destruction by immune-associated complement and antibodies are directly responsible for thrombocytopenia (9, 10). However, in the early stage of COVID-19, platelet consumption results from the formation of microthrombi, which then stimulates the release of new platelets by megakaryocytes from the bone marrow in a system of feedback compensation, resulting in a slight or no reduction in platelet count. In a retrospective, observational study conducted by Barett et al., high immature platelet fraction and increased mean platelet volume were found in patients with COVID-19, indicating an accelerated rate of platelet renewal (11). In other comparative studies, similar increased immature platelet count was shown in patients with COVID-19 (12, 13). For severe and critically ill patients, the consumption of platelets and coagulation factors is massively increased due to platelet activation by cytokine storm, causing severe endothelial damage leading to enhanced contact between platelets and endothelial cells, white blood cells and erythrocytes, which promotes extensive formation of thrombi. When the bone marrow cannot mobilize and release sufficient platelets, exhaustion of platelet levels occurs (14). Thus, low platelet count has been shown to be associated with the severity of COVID-19 (15). The results of a retrospective cohort study showed that 36.2% of COVID-19 patients had low platelet counts (< 150*10^9^/L) on admission. With increasing disease severity, the incidence of low platelet counts increased. And platelet levels were lower in critically ill patients than in severe patients (16). Chen et al. studied low platelet counts that occurred 14 days after symptom onset (known as delayed-phase thrombocytopenia) and showed that 11.8% of patients developed transient delayed-phase thrombocytopenia (17). This could be a combination of the immune system and impaired megakaryocyte growth. Townsend et al. evaluated coagulation markers four months after SARS-CoV-2 infection, and more than 90% of convalescent patients had platelet counts in the normal range (18). Interestingly, a rare complication of adenovirus-based vaccination against SARS-CoV-2, vaccine-induced immune thrombotic thrombocytopenia (VITT), also leads to low platelet counts (19–23). Additionally, in a self-controlled case series study, low platelet counts and an increased risk of thrombotic events were observed following initial vaccination with ChAdOx1 nCoV-19 and BNT162b2 mRNA vaccines (24). The specific pathogenesis in these phenomena is not fully understood, although initial research into VITT suggests that it is mediated through anti-platelet factor 4 antibodies that activate platelets leading to thrombosis and consumptive coagulopathy (25). It may also be related to variant proteins produced in human cells in response to the mRNA carried by the vector (26).

During inflammation, platelets not only coordinate the chemotaxis of leukocytes but also maintain the integrity of the endothelial barrier at the injury site in what is termed “inflammation-related hemostasis”. In fact, evidence has shown that platelets protect the integrity of microcirculation. For example, in mice with acute skin inflammation, bleeding events occurred early and persisted in the platelet-depleted group, while only a few ecchymoses occurred in the normal platelet group (27). Another study found that mice deficient in the platelet-derived angiogenesis protein 1 (ANGPT1) gene had increased microsphere leaks at the site of leukocyte extravasation, with similar results using an anti-glycoprotein Ibα antibody. The authors propose that platelet ANGPT1 interacts with endothelial Tie-2 to prevent passive plasma leakage (28). The permeability of pulmonary microcirculation is of particular concern. The blood-air barrier, where the lungs communicate with the outside world, is highly susceptible to inflammation when pathogens invade, and studies have shown that megakaryocytes exist in large quantities in the pulmonary circulation where they are able to dynamically release platelets in the lungs (29–31). Histological and experimental examination of patients with inflammatory lung disease revealed an increased number of peripheral blood megakaryocytes (32). Postmortem reports of non-survivors of COVID-19 also showed significantly elevated megakaryocyte levels, which may be related to the promotion of thrombopoietin production and upregulation of JAK-STAT signaling by the high inflammatory response (33). At the same time, shortened megakaryopoiesis and retention of megakaryocytes in the lung result in no concomitant increase in platelet count (34). In studies of isolated rabbit and sheep lungs, an artificially low platelet state increased pulmonary microvascular permeability, leading to pulmonary edema and fluid accumulation (35, 36). Pulmonary capillary leakage and low platelet counts are often observed together in critically ill ARDS patients (37, 38). Human platelets have also been observed to reduce vascular endothelial permeability *in vitro* (39). In another ARDS study, the use of the platelet C-type lectin-like 2 (CLEC-2) ligand (podoplanin) protected alveolar-capillary integrity (40). Therefore, the protective effect of platelets on the vascular endothelium may be underappreciated in acute lung infection. Prior to the occurrence of pulmonary capillary leakage, platelets, together with endothelial cells, can protect the vascular wall structure of pulmonary microcirculation in the early immune response to avoid the leakage of blood components into the interstitium and alveolar cavity and to ensure the normal lung function.

In severe and critical COVID-19 patients, the incidence of severe pulmonary inflammation is high, often accompanied by the pathological manifestations of diffuse alveolar injury including the leakage of plasma components into the alveolar cavity (41). At this time, the viral load increases sharply, and abundant immune cells are recruited and activated to release inflammatory mediators and cytokines, which damage alveolar epithelial cells and vascular endothelial cells. Although cytokine storm is an important factor in causing severe pneumonia and pulmonary dysfunction (6, 7, 42), the weakening of vascular endothelial protection caused by the reduction of platelets also contributes. The integrity of the blood vessel wall cannot be maintained, and the permeability of the pulmonary microcirculation is further increased by the combined effects of viruses, inflammation, and increased pulmonary vascular pressure. With the gradual accumulation of fluid, the effective volume of the alveolar space, the tidal volume and vital capacity decrease, worsening dyspnea. Airflow leads to the evaporation of water in the alveolar cavity, causing residual plasma proteins and necrotic alveolar epithelial debris to form a hyaline membrane (43). Even if sufficient oxygen can be ensured, the pulmonary condition cannot be reversed due to the destruction of the lung structure and the reduction of the effective volume of the alveolar space (43, 44).

## Enhanced platelet activity: Thrombogenesis

**Figure 1:**
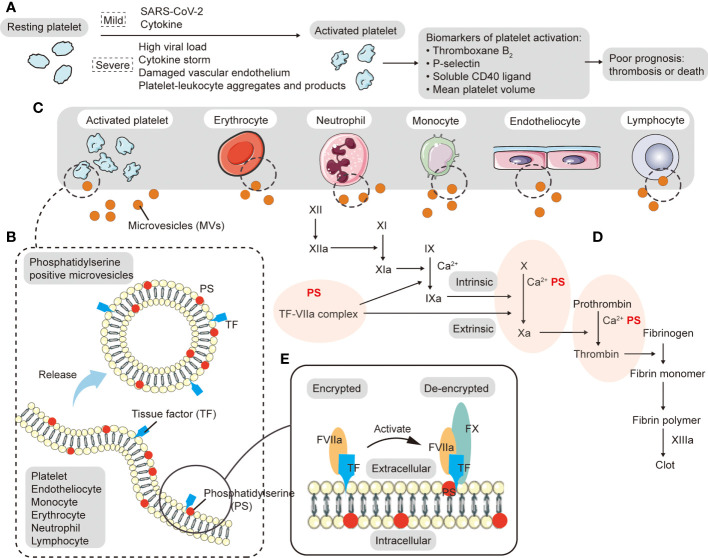
Origin and procoagulant activity of microvesicles (MVs). **(A)** During acute local inflammation, invasive SARS-CoV-2 triggers the immune response in which neutrophils and monocytes are recruited and cytokines activate platelets. When COVID-19 worsens, platelets are easily activated by high viral load, cytokine storm, damaged vascular endothelium, and platelet-leukocyte aggregates, forming widespread (micro-) blood clots in the body, which can lead to poor circulation, hypoxemia and multiple organ failure. Platelet activation markers (such as thromboxane B_2_, p-selectin, soluble CD40 ligand, and mean platelet volume) are associated with increased mortality and the risk of thrombosis. **(B)** MVs are plasma-membrane-derived particles that can be generated by direct budding or fission when cytoskeletal proteins are disrupted. Both tissue factor (TF) and phosphatidylserine (PS) on the surface of MVs can promote the generation of the procoagulant state. **(C)** Various types of cells release MVs in the circulation, including platelets, endothelial cells, red blood cells, neutrophils, lymphocytes, and monocytes, among which platelets are the most important. **(D)** In addition to providing a phospholipid surface for the synthesis of the intrinsic and extrinsic tenase complex (FIXa-FVIIa-Ca^2+^-PS) and prothrombinase complex (FXa-FVa-Ca^2+^-PS), PS can also facilitate the decryption of TF and promote TF-FVIIa complex formation to participate in extrinsic coagulation. The TF-FVIIa complex catalyzes the activation of FX and FIX. **(E)** PS can facilitate the decryption of TF.

Physiological hemostasis is a repair process involving platelets, coagulation cascade, and a variety of factors that form platelet-fibrin complexes locally at the damaged blood vessel site (45). Thrombosis is a disordered pathological process (46, 47). Platelets bind to von Willebrand factor (vWF), which is attached to subcutaneous collagen fibers *via* glycoprotein (GP) Ib/IX/V complexes. Platelet granules are then released. Further, adenosine diphosphate (ADP), thromboxane A_2_ (TXA_2_), 5-hydroxytryptamine, and platelet factor IV are released from the granules. Platelets aggregate due to the action of GP IIb/IIIa, Ca^2+^, and fibrinogen. Under the synergistic effect of thrombin, ADP, TXA_2_, and intrinsic and extrinsic coagulation pathways, platelets aggregate inside the growing thrombus, which locally seals off damaged blood vessels. The risks of thrombosis and embolism are important to consider in pulmonary syndrome. They affect the ventilation/perfusion ratio and cause hypoxia in serious cases. From the acute phase to the recovery phase, platelet activity should be continuously monitored in COVID-19 patients. A single-center cross-sectional study showed that the platelet activation marker soluble CD40 ligand (sCD40L) was significantly higher in intensive care unit (ICU) patients than in non-COVID-19 and non-hospitalized controls (48). Barrett et al. found that in hospitalized patients with COVID-19, platelet activation markers (such as thromboxane B_2_ (TXB_2_), p-selectin, and sCD40L) were associated with increased mortality, with plasma TXB_2_ increasing the risk of thrombosis (**Figure 1A**) (49). Alterations in platelet phenotype can also occur in patients with mild symptoms and may even persist into the recovery phase. McCafferty et al. compared the phenotype of platelets in SARS-CoV-2 positive group and SARS-CoV-2 exposed individuals and found an increase in both PAC1-positive and CD62P-positive platelets (50). The data confirm platelet activation and suggest that patients with mild disease may be at risk for thrombosis during both the acute and convalescent periods. These studies indicate that the risk of thrombotic events may always exist in COVID-19 patients whether with mild or severe disease in the short or long term. The presence of SARS-CoV-2 particles and viral proteins in a significant fraction of platelets has been detected in severe COVID-19 patients, and virus-platelet interactions amplify platelet activation. As part of the defense mechanisms against SARS-CoV-2, platelets recognize intracellular viral components to degrade them *via* autophagosomes and autophagolysosomes (51). Platelets containing viruses may also originate from bone marrow and lung megakaryocytes infected by SARS-CoV-2 and become infectious through being captured by lung macrophages (34). SARS-CoV-2 appears to have the ability to impact mitochondria in platelets and lead to thrombotic or bleeding events depending on the extent of mitochondrial damage (52).

In COVID-19, the classic cause of respiratory dysfunction is local or diffuse pulmonary inflammation. As the disease progresses chest radiographic findings change from patchy shadows in the lateral lung to bilateral pulmonary GGO (53). However, there are also studies reporting that patients with COVID-19 hypoxemia have good lung compliance but abnormal pulmonary blood perfusion (54, 55). In addition, autopsy results showed microthrombi around normal alveoli (9). These results emphasize the importance of thrombogenesis induced by activated platelets. In severe cases, pulmonary (micro)thrombosis occurs, and pulmonary capillary pressure increases, leading to pulmonary hypertension. The pressure difference between the two sides of the blood-air barrier pushes more plasma components into the alveolar cavity. Since the cardiopulmonary system is connected by blood vessels, pulmonary hypertension caused by vascular thrombosis can be transmitted to the heart. At the same time, cytokine storm and endothelial dysfunction also play a synergistic role in the formation of extrapulmonary complications. Of concern is that cardiovascular disease may further aggravate hypoxemia. In the acute phase, the compensatory manifestations of increased heart rate and enhanced myocardial contractility can aggravate myocardial oxygen consumption and accelerate the occurrence of myocardial dysfunction. In the decompensated phase, normal myocardial contraction and relaxation are impaired. With pulmonary circulation congestion and insufficient systemic circulation blood volume, patients may develop systemic ischemia and hypoxia, accompanied by decreased return blood volume.

## Persistent factors promoting platelet-thrombogenesis in COVID-19

### Vascular endothelial injury

Under physiological conditions, vascular endothelial cells have smooth surfaces, which inhibit platelet adhesion and fully cover the underlying collagen fibers. The endothelium releases prostacyclin (PGI_2_) and nitric oxide (NO) to inhibit platelet activation and prevents platelet amplification by NTPDase-1 (46). Elevated levels of endothelial stress products are often detected in the circulating blood of COVID-19 patients. Goshua et al. found that 80% of non-ICU patients had higher concentrations of vWF antigen than normal. ICU patients were associated with further elevated markers of vascular endothelial and platelet activation, including vWF antigen (565% vs. 278%) and soluble P-selectin (15.9 ng/mL vs. 11.2 ng/mL) (48). Another study indicated that among ICU patients with COVID-19, levels of endothelium biomarkers (such as soluble E-selectin, soluble P-selectin, angiogenin (Ang) 2, soluble intercellular adhesion molecule-1, and vWF) were higher in non-survivors (56). Fogarty et al. evaluated patients recovering from COVID-19 and found that vWF antigen, vWF propeptide, factor VIII and soluble thrombomodulin were significantly elevated (57). Persistent endothelial injury may be observed even in patients who are not hospitalized. These results suggest that activation of vascular endothelial cells may persist. In another study, Rauch et al. co-cultured human pulmonary microvascular endothelial cells (HPMVEC) *in vitro* with plasma from patients in acute and convalescent phase and observed that plasma from COVID-19 patients significantly reduced the activity of HPMVEC (58). Among them, the cytotoxicity of ICU patient plasma was the strongest, indicating that infected plasma can trigger microvascular endothelial cell damage.

In respiratory diseases, the alveolar side of the blood-air barrier can initiate the immune response through direct interaction with pathogens, while the vascular endothelial side is often affected by inflammation (59). At first, local damage occurs at the weak part of the blood-air barrier, followed by the aggregation of immune cells and cytokines. Cascading inflammatory reactions form a cytokine storm, which increases vascular endothelial permeability, accelerates the influx of inflammatory factors, and gradually increases the scope of vascular endothelial injury. A series of autopsy results of COVID-19 deaths revealed that viral components, such as SARS-CoV-2 inclusion bodies, were found in vascular endothelial cells. Studies have shown that immunocompromised patients are at risk for virus persistence, co-infection, and reinfection (60, 61), and the virus has the possibility of hiding in the body resulting in long-term symptoms (62). SARS-CoV-2 invades vascular endothelial cells and binds to ACE2 receptors, inducing a procoagulant state. ACE/ACE2 imbalance inhibits the conversion of angiotensin (Ang) II to Ang1-7. Ang II, which is generated by Ang I through ACE, then accumulates, causes vasoconstriction, endothelial activation, and thrombosis, affecting hemodynamic homeostasis (63–66). An experimental model of ACE2 knockout mice promotes vascular inflammation and platelet aggregation, while increased ACE2 can provide protection for the vascular endothelium (65).

After vascular endothelial cells are damaged, the factors (PGI_2_, NO, NTPDase-1), which inhibit platelet activation, aggregation, and expansion, decrease. Exposed basal membrane collagen enhances platelet adhesion by binding with platelet membrane GP Ib/IX/V complex *via* bridging molecule vWF. Numerous studies have shown that high levels of vWF are common in all stages of COVID-19 (48, 56, 57). Damaged endothelium can also promote the coagulation cascade downstream of platelets by expressing tissue factor (TF), entrapping other blood cells to form firm clumps. Moreover, the release of TF pathway inhibitors is reduced, promoting local coagulation. In a baboon model of sepsis, TF-induced coagulation cascades played a key role, and inhibition of TF effectively reduced multiple organ failure and mortality (67). Many studies have also demonstrated that the up-regulation of TF expression is related to the formation of immune thrombosis in COVID-19 (68, 69). A cross-sectional study found that thrombin-antithrombin III complex concentrations and FVIII activity were elevated in 90% of non-ICU patients, and significantly increased in ICU patients (48). From the perspective of hemodynamics, local vasoconstriction and slow blood flow are conducive to platelet adhesion when pulmonary vascular endothelial cells are damaged.

### Inflammation

Pulmonary inflammation is the most common pathological change in COVID-19. In a follow-up study testing COVID-19 patients for 34 inflammatory-related substances, patients with mild symptoms showed higher levels of chemokine RANTES (CCL5), while patients with later-stage disease had significant increases in cytokines such as interleukin (IL) 6 and interferon-γ (IFN-γ) (70). Diffuse alveolar damage can be seen in many patients with severe and critical COVID-19. Inflammatory infiltration of the pulmonary interstitium and deposition of intravascular platelet-fibrin thrombi are important features. In COVID-19, the co-occurrence of inflammation and platelet thrombus on both sides of the blood-air barrier also suggests the close relationship between platelets and the immune system. A retrospective study by Chen et al. found that in severe COVID-19, inflammatory markers (lactate dehydrogenase, C-reactive protein, ferritin, procalcitonin, tumor necrosis factor α (TNF-α), IL-2R, IL-6, and IL-10) were significantly elevated and T-cell subsets (CD4^+^ T cells and CD8^+^ T cells) were reduced (71). Another autopsy study showed interstitial mononuclear inflammatory infiltration bilaterally in the liver and especially the lungs. The results indicated that the concentration of pro-inflammatory CCR6^+^ Th17 increased, enhancing cytotoxicity in CD8^+^ T cells (72). In the whole-body tissue atlas of COVID-19 established by Dorward et al., the lungs presented with diffuse alveolar damage and bronchopneumonia. Patchy vasculitis of the intima of small and medium pulmonary arteries was also seen, mainly infiltrated by MRP8^+^ monocytes and mixed with T cells and macrophages (73). A similar phenomenon was seen in SARS. Histopathology of severe SARS indicated a large number of macrophages in the alveoli and lung interstitium (74). Serological measurements have shown that cytokines (Th1-type interferon, inflammatory cytokines IL-1, IL-6, and IL-12) and chemokines (neutrophil chemokine IL-8, monocyte chemokine protein-1, and Th1 chemokine IFN-γ-inducible protein-10) are significantly increased (75). Accumulation of pathogenic monocytes/macrophages and elevated cytokine/chemokine was also detected in a mouse model of SARS-CoV (76). GGO caused by pulmonary inflammatory exudation is a common imaging manifestation in the acute phase of COVID-19 that can also appear in convalescent patients. In a cohort study of thoracic high-resolution computed tomography, GGO remained the most common symptom in previously hospitalized COVID-19 patients at six months of recovery (5). At another follow-up at 12 months, 76% of the severe group still had GGO (4).

Inflammation interacts with platelets, inducing a vicious cycle (77, 78). Inflammation/immunity is thought to be a potential promoter of platelet activation, causing the transition of normal platelets to a procoagulant phenotype and eventually forming thrombosis. Using *in vitro* experiments, Hottz et al. showed that monocyte subsets with high CD16 and low HLA-DR expression interacted with platelets to promote TF expression and secrete more TNF-α and IL-1β, amplifying inflammation in severe COVID-19 (79). Studies have indicated that toll-like receptor 4 signaling stimulates neutrophils to bind to activated platelets and then release neutrophil extracellular traps (77). Cell-free DNA activates the coagulation cascade; and histone components convert platelets, red blood cells, and endothelial cells to a procoagulant state (80). These need to be noted in COVID-19 patients with bacterial infection or sepsis. Both *in vivo* and *in vitro* experiments have proved that TNF-α/TNF-r1 signal and IL-1 can promote the expression of vascular endothelial vWF, and facilitate platelets adhesion to endothelial cells *via* GP Ib/lX/V. Then it transitions to a hypercoagulable state (81). In addition, inflammatory cytokines can also enhance the coagulation cascade downstream of the platelet-thrombus. For example, IL-1 and TNF-α can stimulate the synthesis of coagulation factors and activate monocytes and endothelial cells (82). Inflammation or cytokine storm can also indirectly promote the production of procoagulant platelets by damaging local or enlarged vascular endothelial cells. In addition, platelets enhance inflammatory responses, such as regulating immune cell function, promoting platelet-neutrophil aggregation, storing immunomodulatory molecules (transforming growth factor-β, IL-1, platelet-derived growth factor, and CC-chemokine ligand 5), and expressing Toll-like receptors to bind microorganisms (83, 84). The role of platelet-mediated vascular inflammation has been demonstrated in atherosclerosis and myocardial infarction (85, 86).

### Hypoxia

Dyspnea and hypoxemia can be used to assess disease progression and prognosis in COVID-19. Shortness of breath and dyspnea are common symptoms at admission. The majority of hospitalized patients present with mild hypoxemia, but some patients do not develop respiratory distress until partial arterial oxygen pressure (PaO_2_) drops to 60 mmHg (or lower). This indicates that even if the clinical symptoms are mild at admission, the patient’s condition may actually be more severe. In a study in Seattle, when patients were admitted, 53% had dyspnea, 86% had an abnormal initial chest X-ray, and 41% developed hypoxia (1). In another retrospective study, mortality was higher in patients with initial symptoms of respiratory distress, and hypoxemia was an independent predictor of in-hospital mortality (87). The long-term effects of SARS-CoV-2 on the lungs require continuous monitoring of the patients’ respiratory symptoms, lung imaging, and lung function tests. Mo et al. analyzed the lung function of discharged COVID-19 survivors. Of those studied, 30.4% of patients with mild pneumonia and 84.2% of patients with severe pneumonia showed impaired diffusion volume (2). A similar pattern was observed in patients 30 days after discharge. In patients discharged after severe disease, lung function was seriously impaired, with a higher proportion of DLCO and imaging abnormalities (3). A comparison of lung function at 6 and 12 months of discharge showed no significant improvement in dyspnea and diffuse lung injury, with 54% of the severe group having diffuse lung injury one year after discharge, compared to 23% of patients with milder illness (4, 5). Pulmonary fibrosis is another important cause of poor pulmonary function in convalescence. Pulmonary fibrosis is closely related to protein deposition in the extracellular stroma and fibroblast migration into muscle cells. Elevated levels of growth factor receptor B1 and respiratory virus infection are involved in this process (5, 88). Hypoxia can lead to inflammation-related gene transcription and aggravate endothelial injury through hypoxia inducible factor (HIF) and NF-Bĸ pathway (89). HIF-1α promotes the production of neutrophil extracellular traps, and cell-free DNA can activate the contact clotting pathway by providing negatively charged surfaces. Histone components can bind to platelets, red blood cells, and endothelial cells and then activate a procoagulant phenotype (80). In a hypoxic rat model, bleeding time was significantly shortened in the hypoxic exposure group. Coagulation tests showed reduced prothrombin time and activated partial thromboplastin time, as well as higher platelet reactivity and stronger interaction with fibrinogen (90). In other pulmonary syndromes such as chronic obstructive pulmonary disease and sleep apnea, hypoxia has been implicated in platelet hyperresponsiveness and aggregation (91, 92). The blood clots cause poor blood flow in the pulmonary microcirculation, so the vicious cycle of hypoxia and thrombosis can exacerbate pulmonary dysfunction.

## Microvesicles (MVs), platelets, and thrombosis

MVs are plasma-membrane-derived particles that can be generated by direct budding or fission when cytoskeletal proteins are disrupted (**Figure 1B**), and they are mainly derived from activated, injured and dead cells. In a high intracellular Ca^2+^ environment, phospholipid translocation enzymes (floppase and flippase) are inactivated. PS is exposed to the outer surface of the cell membrane by scramblase, resulting in a loss of plasma membrane asymmetry and release of MVs (93–95). Various types of cells release MVs in the circulation, including platelets, endothelial cells, red blood cells, neutrophils, lymphocytes, and monocytes (**Figure 1C**) (96, 97). During acute local inflammation, invasive SARS-CoV-2 triggers the immune response in which neutrophils and monocytes are recruited and cytokines activate platelets. With viral clearance and cytokine production, neutrophils, monocytes, lymphocytes, and platelets undergo activation, damage, and death, releasing MVs, while lymphocytes primarily produce killer toxins and promote antibody production. Rausch et al. found that the number of PS^+^ peripheral blood mononuclear cells (PBMCs) in patients within weeks of the initial diagnosis of COVID-19 was significantly higher than that in healthy controls or recovered patients (98). When COVID-19 worsens, platelets are easily activated by high viral load, cytokine storm, damaged vascular endothelium, and platelet-leukocyte aggregates, forming widespread (micro-) blood clots in the body, which can lead to poor circulation, hypoxemia and multiple organ failure (**Figure 1A**). These platelets have the characteristics of large average volume, high activity and short life (as shown by an increased proportion of immature platelets). In response to stimulation by massive amounts of cytokines, platelets release large numbers of platelet extracellular vesicles (pEVs). Such pEVs are the main circulating MVs in prethrombotic and inflammatory diseases (99). Zaid et al. showed that pEVs concentrations were higher in non-severe or severe COVID-19 patients than in healthy controls (100). By grouping surface antigens, Balbi et al. found that in SARS-CoV-2 infection, most MVs originated from either the endothelium or platelets (101). Flaumenhaft et al. demonstrated that megakaryocytes also produce MVs directly, mainly CD41^+^, CD42b^+^ and PS-expressed MVs (102). MVs can also be found in endotheliocytes and erythrocytes that have been damaged in the thrombo-inflammatory environment and then cleared by various immune cells. Similarly elevated levels of MVs have been seen in other lung diseases and viral infections. For example, compared with hydrostatic edema, ARDS had a higher concentration of alveolar epithelial MVs which showed stronger procoagulant effects (103). High levels of pEVs are also seen in other viral infections, such as influenza A and dengue fever. They promote pEVs release through an FcgRIIa-related mechanism and a CLEC-2-dependent manner, respectively (104, 105).

Both TF and PS on the surface of MVs can promote the generation of the procoagulant state (**Figure 1B**), which plays an important role in the activation of the coagulation cascade and the formation of arterial/venous thrombosis. The TF-FVIIa complex catalyzes the activation of FX and FIX. A study analyzed MVs TF activity in COVID-19 patients, and found that SARS-CoV-2 may induce TF^+^ MVs release in moderate and severe disease. Results also indicated that MVs TF activity was correlated with coagulation markers (D-dimer, international normalized ratio, prothrombin, fibrinogen. and antithrombin levels), fibrinolytic levels (plasminase-antifibrinolytic complex), and endothelial activation markers (vWF) (106). In addition to providing a phospholipid surface for the synthesis of the intrinsic pathway factor Xase complex (FIXa-FVIIa-Ca^2+^-PS) and prothrombinase complex (FXa-FVa-Ca^2+^-PS) (**Figure 1D**), PS can also facilitate the decryption of TF and promote TF-FVIIa complex formation to participate in extrinsic coagulation (**Figure 1E**). Studies have shown that high PS content of about 30% enables the TF-FVIIa complex to exert the highest procoagulant activity (107). MVs have been associated with the initial formation of platelet thrombi in many animal models. For example, monocyte-derived TF^+^ MVs were involved in the accumulation of platelet-rich thrombi after laser-induced endothelial injury in mice (108). Another *in vitro* study showed that short duration (2-4 h) exposure to monocyte-derived MVs can lead to PS exposure on the surface of human umbilical vein endothelial cells and induce procoagulant activity and apoptosis of endothelial cells (109). Increased MVs may also predict the occurrence of thrombotic events. One study has shown that compared with healthy controls, non-severe COVID-19 group, and sepsis ICU group, platelet membrane potential depolarization, cytoplasmic Ca^2+^, and PS externalization were significantly higher in COVID-19 ICU patients, and were correlated with organ failure and increased D-Dimer (110). In addition, the frequency of PS^+^ and MVs-bound PBMCs in recovered patients was also statistically significantly higher than that in healthy donors, indicating that PS^+^ MVs^+^ PBMCs may be a predictor of long-term adverse prognosis in COVID-19 (98). In a prospective study conducted by Amabile et al., circulating MVs were an independent predictor of myocardial infarction and stroke in patients with end-stage renal failure (111). Pulmonary thrombotic events appear to accelerate the development of severe conditions. There is a close relationship between MVs, PS, platelets, and coagulation factors, and therefore special attention is needed.

## Treatment

In COVID-19, the most commonly used means of inhibiting hypercoagulability and preventing thrombotic events is anticoagulant therapy, such as unfractionated heparin, low molecular weight heparin, and direct oral anticoagulant drugs. As a part of comprehensive antithrombotic therapy, the ultimate goal of antiplatelet therapy is to control the downstream coagulation cascade by inhibiting platelet adhesion, aggregation, and release so as to inhibit thrombosis. In a multivariate analysis, Santoro et al. found that in-hospital treatment with antiplatelet drugs (e.g., aspirin, clopidogrel, ticlopidine, prasugrel, or ticagrelor) reduces mortality, even in subgroups of patients in ICU (112). Results from a retrospective study showed that patients treated with aspirin had lower cumulative morbidity during hospitalization than those without antiplatelet drugs, whether routinely used in critically ill patients or recommended for all hospitalized patients (113). Another randomized controlled trial indicated a decrease in the incidence of thrombosis (4.6% vs. 5.3%) and a 1.2% improvement in survival in the aspirin group (150 mg daily) compared with the usual care group (114). Some antiplatelet agents also have antiviral, anti-inflammatory, and anti-cytokine storm mitigation effects. For example, aspirin irreversibly inhibits cycloxygenase and inhibits inflammatory effects related to the nuclear transcription of Kappa B (115). P2Y_12_ inhibitors exert anti-inflammatory effects by attenuating cytokine release, platelet-leukocyte interaction, and the formation of neutrophil extracellular traps (116). Under the combined action of anti-platelet and anti-inflammatory treatments, platelets, other blood cells, vascular endothelial, and alveolar epithelial cells are less damaged. The decrease in phosphatidylserine exposure and MVs release inhibits the decryption of tissue factor and the formation of intrinsic and extrinsic tenase and prothrombinase complexes, thus indirectly inhibiting the coagulation cascade triggered by phosphatidylserine and blocking the formation of thrombosis.

As for the controversies about the efficacy of antiplatelet drugs, different conclusions may be drawn based on the bias in clinical trials, such as patients with different disease severity, the phase and duration of drug use, combinations of other drugs, diverse primary outcomes, and so on (**Table 1**). Analysis of a retrospective study showed no association of antiplatelet therapy (aspirin and/or P2Y_12_ receptor antagonists) with survival in hospitalized patients with COVID-19 (119). In a randomized controlled study, in-hospital antiplatelet therapy was less likely to improve organ support-free days within 21 days in critically ill patients, but it improved the probability of survival to discharge (97%) and survival over 90 days (99.7%) (120). Berger et al. focused on non-critically ill hospitalized patients with COVID-19, and found no improvement in the organ support–free days within 21 days and no significant differences in the incidence of thrombotic events or in-hospital deaths between the additional P2Y_12_ inhibitor group and the control group with therapeutic anticoagulation alone (121). Considering that the lifespan of platelets is only 7-9 days, the late intervention of antiplatelet therapy may miss the opportunity to modulate platelet activation and fail to achieve the expected efficacy. Analysis of an observational cohort study showed that patients receiving aspirin (81 mg daily), clopidogrel (75 mg daily), dipyridamole (200 mg BID), ticagrelor (90 mg BID), or prasugrel (10 mg daily) before admission could reduce the incidence of pulmonary embolism and death (117). In another study, patients who used aspirin (median dose: 81 mg; median treatment duration: 6 days) early in treatment (24 hours after admission or seven days before admission) had lower rates of mechanical ventilation (35.7% vs. 48.4%) and ICU admission (38.8% vs. 51.0%) (118). However, Corrochan et al. rejected the efficacy of early antiplatelet agents and found that pre-admission treatment with anticoagulants and antiplatelet agents did not significantly improve mortality and thrombotic events (122). Results in studies on the early use of antiplatelet agents vary widely. In addition to differences in study design, a variety of measures of adverse outcomes, and discussion of single results versus combined results can have an impact. Another reason for emphasizing early treatment is that by inhibiting (micro)thrombosis, hypoxia due to insufficient pulmonary microcirculation can be avoided. At the same time, the pulmonary vascular pressure can be maintained in the normal range to avoid pulmonary edema caused by the large pressure difference between the two sides of the blood-air barrier. Therefore, it can reduce the incidence of lung consolidation and sequela. Currently, in convalescent patients, no guidelines recommend routine use of antiplatelet agents in patients without thrombotic risk factors.

**Table 1:** Summary of studies on antiplatelet therapy in COVID-19.

Studies	Study design	Population	Start time of APT	Name of APT	Experimentalintervention	Comparator intervention	Follow-up time	Adverse prognosis and all-cause mortality
Evidence of improved prognosis								
Santoro 2022 (112)	Prospective cohort study	7824	Prehospital and in‐hospital	Aspirin, clopidogrel, ticlopidine, prasugrel, or ticagrelor	730 patients (9%) received single APT (n=680) or dual APT (n=50) during hospitalization. 66 patients received additional oral anticoagulants, unfractionated or LMWH.	6930 patients with neither anticoagulation therapy nor APT	Until death or hospital discharge	In-hospital APT was associated with lower mortality risk (RR 0.39, 95% CI 0.32-0.48, p<0.01) in a multivariable analysis including cardiovascular risk factors, anticoagulation therapy, and severe clinical presentation.
Meizlish 2021 (113)	Propensity score‐matched, observational study	638	In‐hospital	Aspirin	319 patients who were not on home antiplatelet therapy received in‐hospital aspirin. Aspirin was for critically ill COVID-19 patients before 18 May 2020, and 81 mg aspirin daily for all hospitalized patients after 18 May 2020.	319 patients treated with no in-hospital aspirin	Until death or hospital discharge	(1) On multivariable analysis of propensity score‐matched patients in the aspirin cohort, the use of in‐hospital aspirin was associated with a lower cumulative incidence of in‐hospital death (HR 0.522, 95% CI 0.336-0.812). (2) Among patients admitted after May 18, the cumulative incidence of in-hospital death was significantly lower in the in-hospital aspirin cohort compared with the no antiplatelet therapy group (HR 0.036, 95% CI 0.002-0.576).
RECOVERY Collaborative Group 2022 (114)	Randomized, controlled, open-label, platform trial	14892	In‐hospital	Aspirin	7351 patients were randomly allocated to usual care plus aspirin (150 mg daily by mouth, nasogastric tube, or rectum).	7541 patients treated with usual care alone	28 days	(1) A reduction in thrombotic events (4.6% vs. 5.3%) and an increase in major bleeding events (1.6% vs. 1.0%) were seen in the aspirin group. (2) Aspirin was associated with a small increase in the rate of being discharged alive within 28 days (75% vs. 74%; RR 1.06, 95% CI 1.02–1.10, p=0.0062) but no improvement in 28-day mortality (17% vs. 17%; RR 0.96, 95% CI 0.89–1.04, p=0.35).
Chow 2021 (117)	Propensity score‐matched, observational study	34675	Prehospital and in‐hospital	Aspirin, clopidogrel, dipyridamole, ticagrelor, or prasugrel	6781 (19.6%) patients received prehospital antiplatelet therapy, such as aspirin (81 mg daily), clopidogrel (75 mg daily), dipyridamole (200 mg BID), ticagrelor (90 mg BID), or prasugrel (10 mg daily). Dual antiplatelet therapy was used in 7.4% of patients. Receipt of other therapeutics (such as dexamethasone and remdesivir) was not different between the two groups.	27894 (80.4%) patients were not on antiplatelet therapy at the time of admission. Then, 29.4% of the control group received in‐hospital antiplatelet therapy.	NR	(1) In‐hospital mortality was significantly lower in patients receiving prehospital antiplatelet therapy (18.9% vs. 21.5%, p<0.001), resulting in a 2.6% absolute reduction in mortality (HR, 0.81, 95% CI: 0.76–0.87, p<0.005). (2) In the antiplatelet therapy group, there was a significantly lower rate of pulmonary embolism (2.2% vs. 3.0%, p=0.002) and higher rate of epistaxis (0.9% vs. 0.4%, p<0.001). There was no difference in the rate of other hemorrhagic or thrombotic complications.
Chow 2021 (118)	Retrospective, observational cohort study	412	Within 24 hours of hospital admission or in the 7 days before hospital admission	Aspirin	98 patients (23.7%) received aspirin within 24 hours of admission or 7 days before admission. The median dose of aspirin was 81 mg and the median treatment duration was 6 days (IQR, 3-12 days).	314 patients (76.3%) received no aspirin	NR	(1) After adjusting for confounding variables, aspirin use was independently associated with a reduction in the risk of mechanical ventilation (aHR, 0.56, 95% CI 0.37-0.85, P=0.007), ICU admission (aHR, 0.57, 95% CI 0.38-0.85, P=0.005), and in-hospital mortality (aHR, 0.53, 95% CI 0.31-0.90, P=0.02). (2) There were no differences in major bleeding or overt thrombosis between aspirin cohort and control group.
Evidence of no improved prognosis								
Schrottmaier 2022 (119)	Retrospective study cohort	578	In‐hospital	Aspirin, clopidogrel, or ticagrelor	114 (20.3%) patients received antiplatelet therapy, such as aspirin (100 mg daily), clopidogrel (75 mg BID), ticagrelor (90 mg BID), or a combination thereof. 522 (92.7%) patients were treated with anticoagulation. General inpatients received prophylactic doses, and patients with suspected pulmonary embolism or new thromboembolic events received therapeutic anticoagulation.	Patients with no antiplatelet therapy or no anticoagulant therapy respectively	NR	Analysis of patient’s survival using Kaplan–Meier curves showed no association of antiplatelet therapy (aspirin and/or P2Y_12_ receptor antagonists) with survival, whereas anticoagulation (LMWH and/or NOAC) was associated with increased survival.
REMAP-CAP Investigators 2022 (120)	Randomized clinical trial	1557 critically ill adult patients	In‐hospital	Aspirin, clopidogrel, ticagrelor, or prasugrel	Patients were randomized to receive aspirin (n=565) (75-100 mg once daily) or a P2Y_12_ inhibitor (n=455), such as clopidogrel (75 mg once daily without a loading dose), ticagrelor (60 mg twice daily without a loading dose), prasugrel (a 60-mg loading dose followed by 10 mg daily if aged <75 years and weight ≥60 kg or 5 mg daily if aged ≥75 years or weight <60 kg). All antiplatelet interventions were administered enterally until study day 14 or hospital discharge, whichever occurred first. Patients received concurrent anticoagulation thromboprophylaxis according to standard care.	529 patients with no antiplatelet therapy	90 days	(1) In the critically ill patients treated with antiplatelet agents, the likelihood of improvement in organ support–free days within 21 days was low. The median for organ support–free days was 7 (IQR 1-16) in both the antiplatelet and control groups (aOR 1.02, 95% CI 0.86-1.23, 95.7% posterior probability of futility). (2) The proportions of patients surviving to hospital discharge were 71.5% and 67.9% in the antiplatelet and control groups, respectively (aOR 1.27, 95% CI 0.99-1.62, adjusted absolute difference, 5%, 97% posterior probability of efficacy). (3) Major bleeding occurred in 2.1% and 0.4% of patients in the antiplatelet and control groups (a OR 2.97, 95% CI 1.23-8.28, adjusted absolute risk increase 0.8%, 99.4% probability of harm).
Berger 2022 (121)	Randomized clinical trial	562 non–critically ill patients	In‐hospital	Ticagrelor, prasugrel, or clopidogrel	293 patients were randomized to a therapeutic dose of heparin plus a P2Y_12_ inhibitor for 14 days or until hospital discharge, whichever was sooner. Ticagrelor (60 mg BID), prasugrel (5 mg daily for <75 years old with <60 kg; 30 mg load, 10 mg daily for <75 years old with ≥60 kg), and clopidogrel (300 mg load then 75 mg daily) were allowed. Concomitant baseline therapies included corticosteroids, remdesivir, and IL-6 receptor antagonists.	269 patients treated with a therapeutic dose of heparin and usual care	90 days	(1) Compared with a therapeutic dose of heparin only, additional P2Y_12_ inhibitor did not improve organ support–free days within 21 days during hospitalization (aOR 0.83, 95% CI 0.55-1.25). (2) No significant differences in the incidence of thrombotic events or in-hospital deaths between the P2Y_12_ inhibitor group and the usual care group (6.1% vs. 4.5%; aOR, 1.42, 95% CI 0.64-3.13) were seen.
Corrochano 2022 (122)	Observational study	1612	Prehospital	Aspirin, clopidogrel, prasugrel, ticagrelor, cangrelor, or dipyridamole	Patients were grouped according to treatment received prior to admission. Before admission, 155 (9.6%) patients received anticoagulants, and 308 (19.1%) antiplatelet therapy. Group 1 was comprised of patients receiving chronic anticoagulation with VKA, DOACs, or heparins. Group 2 consisted of patients receiving antiplatelet therapy.	1135 patients (70.4%) received neither anticoagulation nor antiplatelet therapy.	28 days	(1) On the multivariate analysis (adjusted for sex, age and comorbidities), pre-admission treatment with AC (35.5%, OR 1.07, 95% CI 0.70-1.62, p=0.757) or AP (35.7%, OR 1.18, 95% CI 0.84–1.66, p=0.339) did not significant influence mortality rates. (2) Patients on AC (4.5%, OR 0.41, 95% CI 0.18–0.93, p=0.034) had lower ICU admission rates than the control group. (3) The antithrombotic groups (AC or AP) showed no statistically significant differences with the no-antithrombotic therapy group on the incidence rates for thrombotic events.

APT, antiplatelet therapy; LMWH, low molecular weight heparin; RR, risk ratio; CI, confidence interval; HR, hazard ratio; aOR, adjusted odds ratio; IQR, interquartile range; NR, not reported; aHR, adjusted hazard ratio; VKA, vitamin K antagonists; DOACs, direct oral anticoagulants; AC, anticoagulant; AP, antiplatelet agent; ICU, intensive care unit; NOAC, novel oral anticoagulants.

Since the role of anticoagulants in improving hypercoagulability and preventing thrombosis in COVID-19 has been widely recognized, antiplatelet therapy is more often used as a supplementary treatment. Many guidelines recommend therapeutic doses of low molecular weight heparin for hospitalized, non-critically ill patients, and some narrow the patient population based on oxygen needs or bleeding risk. Prophylactic-intensity anticoagulant therapy for critically ill patients has been widely recommended (123–127). The formation of thrombi or extensive microthrombi consumes a large number of coagulation factors and platelets, leading to a hypofibrinolytic state during which antiplatelet or anticoagulation therapy would be less effective. Thus studies that administer therapeutic anticoagulation later in disease progression may not see a significant extension of survival time or improvement in survival rate. Coagulation abnormalities may exist for a long time, from the acute stage to the sequelae stage. We believe that it is of great importance to carry out comprehensive antithrombotic therapy in the early stage of COVID-19 to prevent thrombosis or remove (micro) thrombosis before there is a significant depletion of coagulation factors. If untreated early, hypercoagulation or thrombosis may cause a rapid increase in D-dimer, along with higher levels of fibrinogen, vWF, and P-selectin (14). When entering the period of severe and critical illness, cytokine storms, severe endothelial damage, intrapulmonary and extrapulmonary thrombosis, and consumption of platelets and clotting factors together lead to a significant risk of bleeding. Some studies of long COVID have detected higher D-dimer levels than normal (128). In addition, multiple studies have shown that patients may experience impaired fibrinolytic function during long COVID, with elevated plasminogen activator inhibitor-1 levels and decreased thrombi solubility (129–132). Buonsenso et al. found that anticoagulant therapy improved the quality of life in patients with poor pulmonary microcirculation on SPECT/CT during long COVID, suggesting extensive cell death, multiple tissue injury, and organ failure (133). The above data indicates that the improvement of hypercoagulability is very promising for the improvement of sequelae. However, the current guidelines do not recommend routine prophylactic doses of anticoagulant after discharge, with the exception of the National Institute for Health and Care Excellence (NICE) guidelines which recommend prophylaxis last for at least 7 days after discharge (125). Exposed PS may be another potential target for inhibiting hypercoagulable states. Studies have shown that PS can act as a signal to promote a hypercoagulable state in COVID-19 (98). In addition to being sensitive probes and effective PS blockers, lactadherin and annexin V act as anticoagulants by blocking activation of the clotting cascade *in vitro* and *in vivo*. Lactadherin inhibits intrinsic pathway factor Xase complex and prothrombinase complex by competing with coagulation factors V and VIII for PS binding sites. It also affects the formation of TF-FVIIa complex and inhibits the activation of FII. In contrast, annexin V is unable to inhibit factor Xase complex and has weak inhibition of the TF-FVIIa complex (134, 135). Dasgupta et al. demonstrated lactadherin-mediated clearance of PS^+^ pEVs in the circulation and observed an increase in the number of pEVs and thrombin production of more than 2 times in lactadherin-deficient mice (136). Therefore, the study of improving the procoagulant state by inhibiting PS can be viewed as a new direction for the treatment of COVID-19.

## Discussion

Low platelet counts and high platelet activity have been seen in both mild and severe COVID-19 patients and correlated with disease severity. For survivors, studies have also observed abnormal platelet counts and platelet activity from the acute phase to the recovery phase. This indicates that platelet-related risk factors for exacerbation may persist, which may not only aggravate disease progression and enhance the risk of thrombosis/embolism events and death, but also increase the incidence of sequelae in discharged patients. Therefore, antiplatelet therapy is still very meaningful, even while the efficacy for patients at different stages may vary. It is ideal to control platelet activation at an early stage, which plays a role in relieving the disease, inhibiting the formation of a hypercoagulable state, reducing thrombotic events and mortality, and improving sequelae.

## Author contributions

MX prepared figures and wrote the manuscript. XW, HJ, LL, CW, and YW provided data and comments. JS came up with the project, designed the study, and contributed to successive drafts. VN reviewed this manuscript. All authors read and approved the final manuscript. All authors contributed to the article and approved the submitted version.

## Acknowledgments

We thank all participants for their contribution to our study and the reviewers for the suggestions provided.

## Conflict of interest

The authors declare that the research was conducted in the absence of any commercial or financial relationships that could be construed as a potential conflict of interest.

## Publisher’s note

All claims expressed in this article are solely those of the authors and do not necessarily represent those of their affiliated organizations, or those of the publisher, the editors and the reviewers. Any product that may be evaluated in this article, or claim that may be made by its manufacturer, is not guaranteed or endorsed by the publisher.

## References

[1] Buckner FS McCulloch DJ Atluri V Blain M McGuffin SA Nalla AK. Clinical features and outcomes of 105 hospitalized patients with COVID-19 in Seattle, Washington. Clin Infect Dis (2020) 71(16):2167–73. doi: 10.1093/cid/ciaa632 PMC7314181 32444880

[2] Mo X Jian W Su Z Chen M Peng H Peng P. Abnormal pulmonary function in COVID-19 patients At time of hospital discharge. Eur Respir J (2020) 55(6):2001217. doi: 10.1183/13993003.01217-2020 32381497 PMC7236826

[3] Huang Y Tan C Wu J Chen M Wang Z Luo L. Impact of coronavirus disease 2019 on pulmonary function in early convalescence phase. Respir Res (2020) 21(1):163. doi: 10.1186/s12931-020-01429-6 32600344 PMC7323373

[4] Huang L Yao Q Gu X Wang Q Ren L Wang Y. 1-year outcomes in hospital survivors with COVID-19: A longitudinal cohort study. Lancet (2021) 398(10302):747–58. doi: 10.1016/S0140-6736(21)01755-4 PMC8389999 34454673

[5] Huang C Huang L Wang Y Li X Ren L Gu X. 6-month consequences of COVID-19 in patients discharged from hospital: A cohort study. Lancet (2021) 397(10270):220–32. doi: 10.1016/S0140-6736(20)32656- PMC7833295 33428867

[6] Caricchio R Gallucci M Dass C Zhang X Gallucci S Fleece D. Preliminary predictive criteria for COVID-19 cytokine storm. Ann Rheum Dis (2021) 80(1):88–95. doi: 10.1136/annrheumdis-2020-218323 32978237

[7] Mangalmurti N Hunter CA. Cytokine storms: Understanding COVID-19. Immunity (2020) 53(1):19–25. doi: 10.1016/j.immuni.2020.06.017 32610079 PMC7321048

[8] Iba T Connors JM Levy JH. The coagulopathy, endotheliopathy, and vasculitis of COVID-19. Inflammation Res (2020) 69(12):1181–9. doi: 10.1007/s00011-020-01401-6 PMC7486586 32918567

[9] Magro C Mulvey JJ Berlin D Nuovo G Salvatore S Harp J. Complement associated microvascular injury and thrombosis in the pathogenesis of severe COVID-19 infection: A report of five cases. Transl Res (2020) 220:1–13. doi: 10.1016/j.trsl.2020.04.007 32299776 PMC7158248

[10] Mei H Luo L Hu Y. Thrombocytopenia and thrombosis in hospitalized patients with COVID-19. J Hematol Oncol (2020) 13(1):161. doi: 10.1186/s13045-020-01003-z 33261634 PMC7705847

[11] Barrett TJ Bilaloglu S Cornwell M Burgess HM Virginio VW Drenkova K. Platelets contribute to disease severity in COVID-19. J Thromb Haemost (2021) 19(12):3139–53. doi: 10.1111/jth.15534 PMC8646651 34538015

[12] Cohen A Harari E Cipok M Laish-Farkash A Bryk G Yahud E. Immature platelets in patients hospitalized with covid-19. J Thromb Thromb (2021) 51(3):608–16. doi: 10.1007/s11239-020-02290-6 PMC7526077 32997333

[13] Welder D Jeon-Slaughter H Ashraf B Choi SH Chen W Ibrahim I. Immature platelets as a biomarker for disease severity and mortality in COVID-19 patients. Br J Haematol (2021) 194(3):530–6. doi: 10.1111/bjh.17656 PMC8444912 34132393

[14] Grobler C Maphumulo SC Grobbelaar LM Bredenkamp JC Laubscher GJ Lourens PJ. Covid-19: The rollercoaster of Fibrin(Ogen), d-dimer, Von willebrand factor, p-selectin and their interactions with endothelial cells, platelets and erythrocytes. Int J Mol Sci (2020) 21(14):5168. doi: 10.3390/ijms21145168 PMC7403995 32708334

[15] Yang X Yang Q Wang Y Wu Y Xu J Yu Y. Thrombocytopenia and its association with mortality in patients with COVID-19. J Thromb Haemost (2020) 18(6):1469–72. doi: 10.1111/jth.1484 PMC9906135 32302435

[16] Liao D Zhou F Luo L Xu M Wang H Xia J. Haematological characteristics and risk factors in the classification and prognosis evaluation of COVID-19: A retrospective cohort study. Lancet Haematol (2020) 7(9):e671–8. doi: 10.1016/S2352-3026(20)30217-9 PMC7351397 32659214

[17] Chen W Li Z Yang B Wang P Zhou Q Zhang Z. Delayed-phase thrombocytopenia in patients with coronavirus disease 2019 (COVID-19). Br J Haematol (2020) 190(2):179–84. doi: 10.1111/bjh.16885 PMC7283673 32453877

[18] Townsend L Fogarty H Dyer A Martin-Loeches I Bannan C Nadarajan P. Prolonged elevation of d-dimer levels in convalescent COVID-19 patients is independent of the acute phase response. J Thromb Haemost (2021) 19(4):1064–70. doi: 10.1111/jth.15267 PMC8013297 33587810

[19] Venkatesan P. Do vaccines protect from long COVID? Lancet Respir Med (2022) 10(3):e30. doi: 10.1016/S2213-2600(22)00020-0 35065716 PMC8776281

[20] Ledford H. Do vaccines protect against long COVID? what the data say. Nature (2021) 599(7886):546–8. doi: 10.1038/d41586-021-03495-2 34815580

[21] Pavord S Scully M Hunt BJ Lester W Bagot C Craven B. Clinical features of vaccine-induced immune thrombocytopenia and thrombosis. N Engl J Med (2021) 385(18):1680–9. doi: 10.1056/NEJMoa2109908 PMC10662971 34379914

[22] Sangli S Virani A Cheronis N Vannatter B Minich C Noronha S. Thrombosis with thrombocytopenia after the messenger RNA-1273 vaccine. Ann Intern Med (2021) 174(10):1480–2. doi: 10.7326/L21-0244 PMC8251935 34181446

[23] Greinacher A Thiele T Warkentin TE Weisser K Kyrle PA Eichinger S. Thrombotic thrombocytopenia after ChAdOx1 nCov-19 vaccination. N Engl J Med (2021) 384(22):2092–101. doi: 10.1056/NEJMoa2104840 PMC8095372 33835769

[24] Hippisley-Cox J Patone M Mei XW Saatci D Dixon S Khunti K. Risk of thrombocytopenia and thromboembolism after covid-19 vaccination and SARS-CoV-2 positive testing: Self-controlled case series study. BMJ (2021) 374:n1931. doi: 10.1136/bmj.n1931 34446426 PMC8388189

[25] Scully M Singh D Lown R Poles A Solomon T Levi M. Pathologic antibodies to platelet factor 4 after ChAdOx1 nCoV-19 vaccination. N Engl J Med (2021) 384(23):2202–11. doi: 10.1056/NEJMoa2105385 PMC8112532 33861525

[26] Sadoff J Davis K Douoguih M. Thrombotic thrombocytopenia after Ad26.COV2.S vaccination - response from the manufacturer. N Engl J Med (2021) 384(20):1965–6. doi: 10.1056/NEJMc2106075 PMC8117965 33861522

[27] Ho-Tin-Noé B Demers M Wagner DD. How platelets safeguard vascular integrity. J Thromb Haemost (2011) Suppl 1(Suppl 1):56–65. doi: 10.1111/j.1538-7836.2011.04317.x PMC3229170 21781242

[28] Braun LJ Stegmeyer RI Schäfer K Volkery S Currie SM Kempe B. Platelets docking to vWF prevent leaks during leukocyte extravasation by stimulating tie-2. Blood (2020) 136(5):627–39. doi: 10.1182/blood.2019003442 PMC7413753 32369573

[29] Weyrich AS Zimmerman GA. Platelets in lung biology. Annu Rev Physiol (2013) 75:569–91. doi: 10.1146/annurev-physiol-030212-183752 PMC3670819 23043249

[30] TM SCHEININ AP KOIVUNIEMI. Megakaryocytes in the pulmonary circulation. Blood (1963) 22:82–7. doi: 10.1182/blood.V22.1.82.82 13991543

[31] Ouzegdouh Y Capron C Bauer T Puymirat E Diehl JL Martin JF. The physical and cellular conditions of the human pulmonary circulation enable thrombopoiesis. Exp Hematol (2018) 63:22–27.e3. doi: 10.1016/j.exphem.2018.04.001 29654952

[32] Trowbridge EA Martin JF Slater DN. Evidence for a theory of physical fragmentation of megakaryocytes, implying that all platelets are produced in the pulmonary circulation. Thromb Res (1982) 28(4):461–75. doi: 10.1016/0049-3848(82)90163-3 7164032

[33] Battina HL Alentado VJ Srour EF Moliterno AR Kacena MA. Interaction of the inflammatory response and megakaryocytes in COVID-19 infection. Exp Hematol (2021) 104:32–9. doi: 10.1016/j.exphem.2021.09.005 PMC8459550 34563606

[34] Zhu A Real F Capron C Rosenberg AR Silvin A Dunsmore G. Infection of lung megakaryocytes and platelets by SARS-CoV-2 anticipate fatal COVID-19. Cell Mol Life Sci (2022) 79(7):365. doi: 10.1007/s00018-022-04318-x 35708858 PMC9201269

[35] Pearse DB Brower RG Adkinson NF Jr Sylvester JT. Spontaneous injury in isolated sheep lungs: Role of perfusate leukocytes and platelets. J Appl Physiol (1985) 1989) 66(3):1287–96. doi: 10.1152/jappl.1989.66.3.1287 2708249

[36] Heffner JE Cook JA Halushka PV. Human platelets modulate edema formation in isolated rabbit lungs. J Clin Invest (1989) 84(3):757–64. doi: 10.1172/JCI114233 PMC329716 2527253

[37] Ware LB Matthay MA. The acute respiratory distress syndrome. N Engl J Med (2000) 342(18):1334–49. doi: 10.1056/NEJM200005043421806 10793167

[38] Hui P Cook DJ Lim W Fraser GA Arnold DM. The frequency and clinical significance of thrombocytopenia complicating critical illness: A systematic review. Chest (2011) 139(2):271–8. doi: 10.1378/chest.10-2243 21071526

[39] Middleton EA Weyrich AS Zimmerman GA. Platelets in pulmonary immune responses and inflammatory lung diseases. Physiol Rev (2016) 96(4):1211–59. doi: 10.1152/physrev.00038.2015 PMC6345245 27489307

[40] Lax S Rayes J Wichaiyo S Haining EJ Lowe K Grygielska B. Platelet CLEC-2 protects against lung injury *via* effects of its ligand podoplanin on inflammatory alveolar macrophages in the mouse. Am J Physiol Lung Cell Mol Physiol (2017) 313(6):L1016–29. doi: 10.1152/ajplung.00023.2017 PMC5814702 28839100

[41] Valdebenito S Bessis S Annane D Lorin de la Grandmaison G Cramer-Bordé E Prideaux B. COVID-19 lung pathogenesis in SARS-CoV-2 autopsy cases. Front Immunol (2021) 12:735922. doi: 10.3389/fimmu.2021.735922 34671353 PMC8521087

[42] Yang L Xie X Tu Z Fu J Xu D Zhou Y. The signal pathways and treatment of cytokine storm in COVID-19. Signal Transduct Target Ther (2021) 6(1):255. doi: 10.1038/s41392-021-00679-0 34234112 PMC8261820

[43] Archer SL Sharp WW Weir EK. Differentiating COVID-19 pneumonia from acute respiratory distress syndrome and high altitude pulmonary edema: Therapeutic implications. Circulation (2020) 142(2):101–4. doi: 10.1161/CIRCULATIONAHA.120.047915 PMC7363563 32369390

[44] Gattinoni L Chiumello D Caironi P Busana M Romitti F Brazzi L. COVID-19 pneumonia: Different respiratory treatments for different phenotypes? Intensive Care Med (2020) 46(6):1099–102. doi: 10.1007/s00134-020-06033-2 PMC7154064 32291463

[45] Versteeg HH Heemskerk JW Levi M Reitsma PH. New fundamentals in hemostasis. Physiol Rev (2013) 93(1):327–58. doi: 10.1152/physrev.00016.2011 23303912

[46] Davì G Patrono C. Platelet activation and atherothrombosis. N Engl J Med (2007) 357(24):2482–94. doi: 10.1056/NEJMra07101 18077812

[47] Mackman N. Triggers, targets and treatments for thrombosis. Nature (2008) 451(7181):914–8. doi: 10.1038/nature06797 PMC2848509 18288180

[48] Goshua G Pine AB Meizlish ML Chang CH Zhang H Bahel P. Endotheliopathy in COVID-19-Associated coagulopathy: Evidence from a single-centre, cross-sectional study. Lancet Haematol (2020) 7(8):e575–82. doi: 10.1016/S2352-3026(20)30216- PMC7326446 32619411

[49] Barrett TJ Lee AH Xia Y Lin LH Black M Cotzia P. Platelet and vascular biomarkers associate with thrombosis and death in coronavirus disease. Circ Res (2020) 127(7):945–7. doi: 10.1161/CIRCRESAHA.120.317803 PMC7478197 32757722

[50] McCafferty C Van Den Helm S Letunica N Attard C Karlaftis V Cai T. Increased platelet activation in SARS-CoV-2 infected non-hospitalised children and adults, and their household contacts. Br J Haematol (2021) 195(1):90–4. doi: 10.1111/bjh.17629 PMC8239564 34101171

[51] Garcia C Au Duong J Poëtte M Ribes A Payre B Mémier V. Platelet activation and partial desensitization are associated with viral xenophagy in patients with severe COVID-19. Blood Adv (2022) 6(13):3884–98. doi: 10.1182/bloodadvances.2022007143 PMC9068266 35789374

[52] Alarabi AB Mohsen A Mizuguchi K Alshbool FZ Khasawneh FT. Co-Expression analysis to identify key modules and hub genes associated with COVID-19 in platelets. BMC Med Genomics (2022) 15(1):83. doi: 10.1186/s12920-022-01222-y 35421970 PMC9008611

[53] Oudkerk M Büller HR Kuijpers D van Es N Oudkerk SF McLoud T. Diagnosis, prevention, and treatment of thromboembolic complications in COVID-19: Report of the national institute for public health of the Netherlands. Radiology (2020) 297(1):E216–22. doi: 10.1148/radiol.2020201629 PMC7233406 32324101

[54] Bhatia P Mohammed S. Severe hypoxemia in early COVID-19 pneumonia. Am J Respir Crit Care Med (2020) 202(4):621–2. doi: 10.1164/rccm.202004-1313LE PMC7427389 32579023

[55] Gattinoni L Coppola S Cressoni M Busana M Rossi S Chiumello D. COVID-19 does not lead to a “Typical” acute respiratory distress syndrome. Am J Respir Crit Care Med (2020) 201(10):1299–300. doi: 10.1164/rccm.202003-0817LE PMC7233352 32228035

[56] Vassiliou AG Keskinidou C Jahaj E Gallos P Dimopoulou I Kotanidou A. ICU Admission levels of endothelial biomarkers as predictors of mortality in critically ill COVID-19 patients. Cells (2021) 10(1):186. doi: 10.3390/cells10010186 33477776 PMC7832393

[57] Fogarty H Townsend L Morrin H Ahmad A Comerford C Karampini E. Persistent endotheliopathy in the pathogenesis of long COVID syndrome. J Thromb Haemost (2021) 19(10):2546–53. doi: 10.1111/jth.15490 PMC8420256 34375505

[58] Rauch A Dupont A Goutay J Caplan M Staessens S Moussa M. Endotheliopathy is induced by plasma from critically ill patients and associated with organ failure in severe COVID-19. Circulation (2020) 142(19):1881–4. doi: 10.1161/CIRCULATIONAHA.120.050907 PMC7643783 32970476

[59] Radeva MY Waschke J. Mind the gap: Mechanisms regulating the endothelial barrier. Acta Physiol (Oxf) (2018) 222(1):e12860. doi: 10.1111/apha.12860 28231640

[60] Tarhini H Recoing A Bridier-Nahmias A Rahi M Lambert C Martres P. Long-term severe acute respiratory syndrome coronavirus 2 (SARS-CoV-2) infectiousness among three immunocompromised patients: From prolonged viral shedding to SARS-CoV-2 superinfection. J Infect Dis (2021) 223(9):1522–7. doi: 10.1093/infdis/jiab075 PMC7928754 33556961

[61] Wajnberg A Amanat F Firpo A Altman DR Bailey MJ Mansour M. Robust neutralizing antibodies to SARS-CoV-2 infection persist for months. Science (2020) 370(6521):1227–30. doi: 10.1126/science.abd772 PMC7810037 33115920

[62] de Melo GD Lazarini F Levallois S Hautefort C Michel V Larrous F. COVID-19-Related anosmia is associated with viral persistence and inflammation in human olfactory epithelium and brain infection in hamsters. Sci Transl Med (2021) 13(596):eabf8396. doi: 10.1126/scitranslmed.abf839 33941622 PMC8158965

[63] Maiuolo J Mollace R Gliozzi M Musolino V Carresi C Paone S. The contribution of endothelial dysfunction in systemic injury subsequent to SARS-CoV-2 infection. Int J Mol Sci (2020) 21(23):9309. doi: 10.3390/ijms21239309 PMC7730352 33291346

[64] Teuwen LA Geldhof V Pasut A Carmeliet P. COVID-19: the vasculature unleashed. Nat Rev Immunol (2020) 20(7):389–91. doi: 10.1038/s41577-020-0343-0 PMC7240244 32439870

[65] Thomas MC Pickering RJ Tsorotes D Koitka A Sheehy K Bernardi S. Genetic ACE2 deficiency accentuates vascular inflammation and atherosclerosis in the ApoE knockout mouse. Circ Res (2010) 107(7):888–97. doi: 10.1161/CIRCRESAHA.110.219279 20671240

[66] Kuba K Imai Y Rao S Gao H Guo F Guan B. A crucial role of angiotensin converting enzyme 2 (ACE2) in SARS coronavirus-induced lung injury. Nat Med (2005) 11(8):875–9. doi: 10.1038/nm1267 PMC7095783 16007097

[67] Creasey AA Chang AC Feigen L Wün TC Taylor FB Jr Hinshaw LB. Tissue factor pathway inhibitor reduces mortality from escherichia coli septic shock. J Clin Invest (1993) 91(6):2850–60. doi: 10.1172/JCI116529 PMC443354 8514893

[68] Hottz ED Azevedo-Quintanilha IG Palhinha L Teixeira L Barreto EA Pão CRR. Platelet activation and platelet-monocyte aggregate formation trigger tissue factor expression in patients with severe COVID-19. Blood (2020) 136(11):1330–41. doi: 10.1182/blood.2020007252 PMC7483437 32678428

[69] Skendros P Mitsios A Chrysanthopoulou A Mastellos DC Metallidis S Rafailidis P. Complement and tissue factor-enriched neutrophil extracellular traps are key drivers in COVID-19 immunothrombosis. J Clin Invest (2020) 130(11):6151–7. doi: 10.1172/JCI14137 PMC7598040 32759504

[70] Zhao Y Qin L Zhang P Li K Liang L Sun J. Longitudinal COVID-19 profiling associates IL-1RA and IL-10 with disease severity and RANTES with mild disease. JCI Insight (2020) 5(13):e139834. doi: 10.1172/jci.insight.139834 PMC7406242 32501293

[71] Chen G Wu D Guo W Cao Y Huang D Wang H. Clinical and immunological features of severe and moderate coronavirus disease 2019. J Clin Invest (2020) 130(5):2620–9. doi: 10.1172/JCI137244 PMC7190990 32217835

[72] Xu Z Shi L Wang Y Zhang J Huang L Zhang C. Pathological findings of COVID-19 associated with acute respiratory distress syndrome. Lancet Respir Med (2020) 8(4):420–2. doi: 10.1016/S2213-2600(20)30076-X PMC7164771 32085846

[73] Dorward DA Russell CD Um IH Elshani M Armstrong SD Penrice-Randal R. Tissue-specific immunopathology in fatal COVID-19. Am J Respir Crit Care Med (2021) 203(2):192–201. doi: 10.1164/rccm.202008-3265OC 33217246 PMC7874430

[74] Nicholls JM Poon LL Lee KC Ng WF Lai ST Leung CY. Lung pathology of fatal severe acute respiratory syndrome. Lancet (2003) 361(9371):1773–8. doi: 10.1016/s0140-6736(03)13413-7 PMC7112492 12781536

[75] Wong CK Lam CW Wu AK Ip WK Lee NL Chan IH. Plasma inflammatory cytokines and chemokines in severe acute respiratory syndrome. Clin Exp Immunol (2004) 136(1):95–103. doi: 10.1111/j.1365-2249.2004.02415.x 15030519 PMC1808997

[76] Channappanavar R Fehr AR Vijay R Mack M Zhao J Meyerholz DK. Dysregulated type I interferon and inflammatory monocyte-macrophage responses cause lethal pneumonia in SARS-CoV-Infected mice. Cell Host Microbe (2016) 19(2):181–93. doi: 10.1016/j.chom.2016.01.007 PMC4752723 26867177

[77] Delvaeye M Conway EM. Coagulation and innate immune responses: Can we view them separately? Blood (2009) 114(12):2367–74. doi: 10.1182/blood-2009-05-199208 19584396

[78] Qu Z Chaikof EL. Interface between hemostasis and adaptive immunity. Curr Opin Immunol (2010) 22(5):634–42. doi: 10.1016/j.coi.2010.08.017 PMC3428061 20932735

[79] Hottz ED Martins-Gonçalves R Palhinha L Azevedo-Quintanilha IG de Campos MM Sacramento CQ. Platelet-monocyte interaction amplifies thromboinflammation through tissue factor signaling in COVID-19. Blood Adv (2022) 6(17):5085–99. doi: 10.1182/bloodadvances.2021006680 PMC9015715 35420680

[80] Noubouossie DF Reeves BN Strahl BD Key NS. Neutrophils: Back in the thrombosis spotlight. Blood (2019) 133(20):2186–97. doi: 10.1182/blood-2018-10-862243 PMC7218731 30898858

[81] Nishimura S Manabe I Nagasaki M Kakuta S Iwakura Y Takayama N. *In vivo* imaging visualizes discoid platelet aggregations without endothelium disruption and implicates contribution of inflammatory cytokine and integrin signaling. Blood (2012) 119(8):e45–56. doi: 10.1182/blood-2011-09-381400 PMC3351094 22096246

[82] Pober JS Sessa WC. Evolving functions of endothelial cells in inflammation. Nat Rev Immunol (2007) 7(10):803–15. doi: 10.1038/nri2171 17893694

[83] Semple JW Italiano JE Jr Freedman J. Platelets and the immune continuum. Nat Rev Immunol (2011) 11(4):264–74. doi: 10.1038/nri2956 21436837

[84] Koupenova M Clancy L Corkrey HA Freedman JE. Circulating platelets as mediators of immunity, inflammation, and thrombosis. Circ Res (2018) 122(2):337–51. doi: 10.1161/CIRCRESAHA.117.310795 PMC5777300 29348254

[85] Lindemann S Krämer B Seizer P Gawaz M. Platelets, inflammation and atherosclerosis. J Thromb Haemost (2007) 5 (Suppl 1):203–11. doi: 10.1111/j.1538-7836.2007.02517.x 17635728

[86] Morrell CN Pariser DN Hilt ZT Vega Ocasio D. The platelet Napoleon complex-small cells, but big immune regulatory functions. Annu Rev Immunol (2019) 37:125–44. doi: 10.1146/annurev-immunol-042718-041607 30485751

[87] Xie J Covassin N Fan Z Singh P Gao W Li G. Association between hypoxemia and mortality in patients with COVID-19. Mayo Clin Proc (2020) 95(6):1138–47. doi: 10.1016/j.mayocp.2020.04.006 PMC7151468 32376101

[88] Cueto-Robledo G Porres-Aguilar M Puebla-Aldama D Barragán-Martínez MDP Jurado-Hernández MY García-César M. Severe pulmonary hypertension: An important sequel after severe post-acute COVID-19 pneumonia. Curr Probl Cardiol (2022) 47(3):101004. doi: 10.1016/j.cpcardiol.2021.101004 34601005 PMC8482545

[89] McElvaney OJ McEvoy NL McElvaney OF Carroll TP Murphy MP Dunlea DM. Characterization of the inflammatory response to severe COVID-19 illness. Am J Respir Crit Care Med (2020) 202(6):812–21. doi: 10.1164/rccm.202005-1583OC PMC7491404 32584597

[90] Tyagi T Ahmad S Gupta N Sahu A Ahmad Y Nair V. Altered expression of platelet proteins and calpain activity mediate hypoxia-induced prothrombotic phenotype. Blood (2014) 123(8):1250–60. doi: 10.1182/blood-2013-05-501924 24297866

[91] Sanner BM Konermann M Tepel M Groetz J Mummenhoff C Zidek W. Platelet function in patients with obstructive sleep apnoea syndrome. Eur Respir J (2000) 16(4):648–52. doi: 10.1034/j.1399-3003.2000.16d14.x 11106207

[92] Rångemark C Hedner JA Carlson JT Gleerup G Winther K. Platelet function and fibrinolytic activity in hypertensive and normotensive sleep apnea patients. Sleep (1995) 18(3):188–94. doi: 10.1093/sleep/18.3.188 7610315

[93] Bevers EM Williamson PL. Getting to the outer leaflet: Physiology of phosphatidylserine exposure at the plasma membrane. Physiol Rev (2016) 96(2):605–45. doi: 10.1152/physrev.00020.2015 26936867

[94] Yu SL Gan XG Huang JM Cao Y Wang YQ Pan SH. Oxalate impairs aminophospholipid translocase activity in renal epithelial cells *Via* oxidative stress: Implications for calcium oxalate urolithiasis. J Urol (2011) 186(3):1114–20. doi: 10.1016/j.juro.2011.04.106 21784463

[95] Hugel B Martínez MC Kunzelmann C Freyssinet JM. Membrane microparticles: Two sides of the coin. Physiol (Bethesda) (2005) 20:22–7. doi: 10.1152/physiol.00029.2004 15653836

[96] Morel O Toti F Hugel B Bakouboula B Camoin-Jau L Dignat-George F. Procoagulant microparticles: Disrupting the vascular homeostasis equation? Arterioscler Thromb Vasc Biol (2006) 26(12):2594–604. doi: 10.1161/01.ATV.0000246775.14471.26 16990554

[97] Owens AP 3rd Mackman N. Microparticles in hemostasis and thrombosis. Circ Res (2011) 108(10):1284–97. doi: 10.1161/CIRCRESAHA.110.233056 PMC3144708 21566224

[98] Rausch L Lutz K Schifferer M Winheim E Gruber R Oesterhaus EF. Binding of phosphatidylserine-positive microparticles by pbmcs classifies disease severity in COVID-19 patients. J Extracell Vesicles (2021) 10(14):e12173. doi: 10.1002/jev2.12173 34854246 PMC8636722

[99] Burnouf T Goubran HA Chou ML Devos D Radosevic M. Platelet microparticles: Detection and assessment of their paradoxical functional roles in disease and regenerative medicine. Blood Rev (2014) 28(4):155–66. doi: 10.1016/j.blre.2014.04.002 24826991

[100] Zaid Y Puhm F Allaeys I Naya A Oudghiri M Khalki L. Platelets can associate with SARS-CoV-2 RNA and are hyperactivated in COVID-19. Circ Res (2020) 127(11):1404–18. doi: 10.1161/CIRCRESAHA.120.31770 PMC7641188 32938299

[101] Balbi C Burrello J Bolis S Lazzarini E Biemmi V Pianezzi E. Circulating extracellular vesicles are endowed with enhanced procoagulant activity in SARS-CoV-2 infection. EBioMedicine (2021) 67:103369. doi: 10.1016/j.ebiom.2021.103369 33971404 PMC8104913

[102] Flaumenhaft R Dilks JR Richardson J Alden E Patel-Hett SR Battinelli E. Megakaryocyte-derived microparticles: Direct visualization and distinction from platelet-derived microparticles. Blood (2009) 113(5):1112–21. doi: 10.1182/blood-2008-06-163832 PMC2635076 18802008

[103] Bastarache JA Fremont RD Kropski JA Bossert FR Ware LB. Procoagulant alveolar microparticles in the lungs of patients with acute respiratory distress syndrome. Am J Physiol Lung Cell Mol Physiol (2009) 297(6):L1035–41. doi: 10.1152/ajplung.00214.2009 PMC2793184 19700643

[104] Boilard E Paré G Rousseau M Cloutier N Dubuc I Lévesque T. Influenza virus H1N1 activates platelets through FcΓRIIA signaling and thrombin generation. Blood (2014) 123(18):2854–63. doi: 10.1182/blood-2013-07-515536 24665136

[105] Sung PS Huang TF Hsieh SL. Extracellular vesicles from CLEC2-activated platelets enhance dengue virus-induced lethality *Via* CLEC5A/TLR2. Nat Commun (2019) 10(1):2402. doi: 10.1038/s41467-019-10360-4 31160588 PMC6546763

[106] Rosell A Havervall S von Meijenfeldt F Hisada Y Aguilera K Grover SP. Patients with COVID-19 have elevated levels of circulating extracellular vesicle tissue factor activity that is associated with severity and mortality-brief report. Arterioscler Thromb Vasc Biol (2021) 41(2):878–82. doi: 10.1161/ATVBAHA.120.315547 PMC7837685 33267656

[107] Neuenschwander PF Bianco-Fisher E Rezaie AR Morrissey JH. Phosphatidylethanolamine augments factor viia-tissue factor activity: Enhancement of sensitivity to phosphatidylserine. Biochemistry (1995) 34(43):13988–93. doi: 10.1021/bi00043a004 7577996

[108] Falati S Gross P Merrill-Skoloff G Furie BC Furie B. Real-time *In vivo* imaging of platelets, tissue factor and fibrin during arterial thrombus formation in the mouse. Nat Med (2002) 8(10):1175–81. doi: 10.1038/nm782 12244306

[109] Aharon A Tamari T Brenner B. Monocyte-derived microparticles and exosomes induce procoagulant and apoptotic effects on endothelial cells. Thromb Haemost (2008) 100(5):878–85. doi: 10.1160/th07-11-0691 18989533

[110] Althaus K Marini I Zlamal J Pelzl L Singh A Häberle H. Antibody-induced procoagulant platelets in severe COVID-19 infection. Blood (2021) 137(8):1061–71. doi: 10.1182/blood.2020008762 PMC7791311 33512415

[111] Amabile N Guérin AP Tedgui A Boulanger CM London GM. Predictive value of circulating endothelial microparticles for cardiovascular mortality in end-stage renal failure: A pilot study. Nephrol Dial Transplant (2012) 27(5):1873–80. doi: 10.1093/ndt/gfr573 22036944

[112] Santoro F Nuñez-Gil IJ Vitale E Viana-Llamas MC Reche-Martinez B Romero-Pareja R. Antiplatelet therapy and outcome in COVID-19: the health outcome predictive evaluation registry. Heart (2022) 108(2):130–6. doi: 10.1136/heartjnl-2021-319552 PMC8494537 34611045

[113] Meizlish ML Goshua G Liu Y Fine R Amin K Chang E. Intermediate-dose anticoagulation, aspirin, and in-hospital mortality in COVID-19: A propensity score-matched analysis. Am J Hematol (2021) 96(4):471–9. doi: 10.1002/ajh.26102 PMC8013588 33476420

[114] Collaborative Group RECOVERY. Aspirin in patients admitted to hospital with COVID-19 (RECOVERY): A randomised, controlled, open-label, platform trial. Lancet (2022) 399(10320):143–51. doi: 10.1016/S0140-6736(21)01825-0 PMC8598213 34800427

[115] Kopp E Ghosh S. Inhibition of NF-kappa b by sodium salicylate and aspirin. Science (1994) 265(5174):956–9. doi: 10.1126/science.8052854 8052854

[116] Mansour A Bachelot-Loza C Nesseler N Gaussem P Gouin-Thibault I. P2Y12 inhibition beyond thrombosis: Effects on inflammation. Int J Mol Sci (2020) 21(4):1391. doi: 10.3390/ijms21041391 PMC7073040 32092903

[117] Chow JH Yin Y Yamane DP Davison D Keneally RJ Hawkins K. Association of prehospital antiplatelet therapy with survival in patients hospitalized with COVID-19: A propensity score-matched analysis. J Thromb Haemost (2021) 19(11):2814–24. doi: 10.1111/jth.15517 PMC8646433 34455688

[118] Chow JH Khanna AK Kethireddy S Yamane D Levine A Jackson AM. Aspirin use is associated with decreased mechanical ventilation, intensive care unit admission, and in-hospital mortality in hospitalized patients with coronavirus disease 2019. Anesth Analg (2021) 132(4):930–41. doi: 10.1213/ANE.0000000000005292 33093359

[119] Schrottmaier WC Pirabe A Pereyra D Heber S Hackl H Schmuckenschlager A. Platelets and antiplatelet medication in COVID-19-Related thrombotic complications. Front Cardiovasc Med (2022) 8:802566. doi: 10.3389/fcvm.2021.802566 35141292 PMC8818754

[120] REMAP-CAP Writing Committee for the REMAP-CAP Investigators Bradbury CA Lawler PR Stanworth SJ McVerry BJ McQuilten Z. Effect of antiplatelet therapy on survival and organ support-free days in critically ill patients with COVID-19: A randomized clinical trial. JAMA (2022) 327(13):1247–59. doi: 10.1001/jama.2022.2910 PMC8941448 35315874

[121] Berger JS Kornblith LZ Gong MN Reynolds HR Cushman M Cheng Y. Effect of P2Y12 inhibitors on survival free of organ support among non-critically ill hospitalized patients with COVID-19: A randomized clinical trial. JAMA (2022) 327(3):227–36. doi: 10.1001/jama.2021.23605 PMC8767444 35040887

[122] Corrochano M Acosta-Isaac R Mojal S Miqueleiz S Rodriguez D Quijada-Manuitt MÁ. Impact of pre-admission antithrombotic therapy on disease severity and mortality in patients hospitalized for COVID-19. J Thromb Thromb (2022) 53(1):96–102. doi: 10.1007/s11239-021-02507-2 PMC8210515 34138399

[123] Moores LK Tritschler T Brosnahan S Carrier M Collen JF Doerschug K. Thromboprophylaxis in patients with COVID-19: A brief update to the CHEST guideline and expert panel report. Chest (2022) 162(1):213–25. doi: 10.1016/j.chest.2022.02.006 PMC8839802 35167861

[124] World Health Organization. Living guidance for clinical management of COVID-19 (2021). Available at: https://www.who.int/publications/i/item/WHO-2019-nCoV-clinical-2021-2. 37184163

[125] The National Institute for Health and Care Excellence. COVID-19 rapid guideline: managing COVID-19 (2022). Available at: https://www.nice.org.uk/guidance/ng191/chapter/Recommendations. 34181371

[126] American Society of Hematology. ASH guidelines on use of anticoagulation in patients with COVID-19 (2022). Available at: https://www.hematology.org/education/clinicians/guidelines-and-quality-care/clinical-practice-guidelines/venous-thromboembolism-guidelines/ash-guidelines-on-use-of-anticoagulation-in-patients-with-covid-19.

[127] National Institutes of Health. Coronavirus disease 2019 (COVID-19) treatment guideines (2022). Available at: https://www.covid19treatmentguidelines.nih.gov. 34003615

[128] Pasini E Corsetti G Romano C Scarabelli TM Chen-Scarabelli C Saravolatz L. Serum metabolic profile in patients with long-covid (PASC) syndrome: Clinical implications. Front Med (Lausanne) (2021) 8:714426. doi: 10.3389/fmed.2021.714426 34368201 PMC8339407

[129] Kruse JM Magomedov A Kurreck A Münch FH Koerner R Kamhieh-Milz J. Thromboembolic complications in critically ill COVID-19 patients are associated with impaired fibrinolysis. Crit Care (2020) 24(1):676. doi: 10.1186/s13054-020-03401-8 33287877 PMC7719734

[130] Meizoso JP Moore HB Moore EE. Fibrinolysis shutdown in COVID-19: Clinical manifestations, molecular mechanisms, and therapeutic implications. J Am Coll Surg (2021) 232(6):995–1003. doi: 10.1016/j.jamcollsurg.2021.02.019 33766727 PMC7982779

[131] Bachler M Bösch J Stürzel DP Hell T Giebl A Ströhle M. Impaired fibrinolysis in critically ill COVID-19 patients. Br J Anaesth (2021) 126(3):590–8. doi: 10.1016/j.bja.2020.12.010 PMC7833514 33422287

[132] Nougier C Benoit R Simon M Desmurs-Clavel H Marcotte G Argaud L. Hypofibrinolytic state and high thrombin generation may play a major role in SARS-COV2 associated thrombosis. J Thromb Haemost (2020) 18(9):2215–9. doi: 10.1111/jth.15016 PMC7405476 32668058

[133] Couzin-Frankel J. Clues to long COVID. Science (2022) 376(6599):1261–5. doi: 10.1126/science.add4297 35709281

[134] Shi J Gilbert GE. Lactadherin inhibits enzyme complexes of blood coagulation by competing for phospholipid-binding sites. Blood (2003) 101(7):2628–36. doi: 10.1182/blood-2002-07-1951 12517809

[135] Shi J Pipe SW Rasmussen JT Heegaard CW Gilbert GE. Lactadherin blocks thrombosis and hemostasis *In vivo*: Correlation with platelet phosphatidylserine exposure. J Thromb Haemost (2008) 6(7):1167–74. doi: 10.1111/j.1538-7836.2008.03010.x 18485093

[136] Dasgupta SK Abdel-Monem H Niravath P Le A Bellera RV Langlois K. Lactadherin and clearance of platelet-derived microvesicles. Blood (2009) 113(6):1332–9. doi: 10.1182/blood-2008-07-167148 PMC2637196 19023116

